# Lactate score predicts survival, immune cell infiltration and response to immunotherapy in breast cancer

**DOI:** 10.3389/fgene.2022.943849

**Published:** 2022-08-15

**Authors:** Ting-Ting Yin, Meng-Xing Huang, Fei Wang, Yi-Hua Jiang, Jie Long, Liang Li, Jie Cao

**Affiliations:** ^1^ Department of General Surgery, Guangzhou Digestive Disease Center, the Second Affiliated Hospital, School of Medicine, South China University of Technology, Guangzhou, China; ^2^ Department of General Surgery, Guangzhou Digestive Disease Center, Guangzhou First People’s Hospital, South China University of Technology, Guangzhou, China; ^3^ Chronic Disease Laboratory, School of Medicine, South China University of Technology, Guangzhou, China; ^4^ Department of Oncology, Guangdong Provincial People’s Hospital, Guangdong Academy of Medical Sciences, Guangzhou, China; ^5^ Guangdong Cardiovascular Institute, Guangdong Provincial People’s Hospital, Guangdong Academy of Medical Sciences, Guangzhou, China; ^6^ Medical Research Center, Guangdong Provincial People’s Hospital, Guangdong Academy of Medical Sciences, Guangzhou, China

**Keywords:** breast cancer, lactate score, tumor microenvironment, immunotherapy, chemotherapy

## Abstract

**Background:** Tumor-derived lactate can modulate the function of infiltrating immune cells to establish an immunosuppressive microenvironment that favors tumor progression. However, possible effects of lactate-related genes (LRGs) on the tumor microenvironment (TME) of breast cancer (BRCA) are still unknown.

**Methods:** LRGs were comprehensively screened from lactate metabolism-related pathways. We correlated the expression of these LRGs with immune cell infiltrating characteristics in the TME and clinicopathological features of patients. We also established a lactate score for quantifying lactate metabolism patterns of cancers and to predict of recurrence-free survival (RFS).

**Results:** We successfully constructed a lactate score that was an independent prognostic factor in BRCA. A low lactate score, which was associated with immune activation with increased CD8^+^ T cells infiltration levels, indicated an inflamed TME. Consistently, higher expression levels of inhibitory immune checkpoints, including PD-L1, LAG3, CTLA4, and TIM3, as observed from high lactate score subgroup, suggested an immune-desert phenotype as well as poor prognosis. Moreover, a low lactate score predicted the increased chemotherapeutic drug sensitivity and enhanced anti-PD-1 immunotherapy responses.

**Conclusion:** The present study analyzed the potential roles of LRGs in the TME diversity and prognosis. These results will help to improve our understanding of the characteristics of TME immune cell infiltration and guide the development of more effective immunotherapy strategies.

## Introduction

Metabolic reprogramming is a key hallmark of cancer (Hanahan and Weinberg, 2011). Aerobic glycolysis, called “Warburg effect” as well, has been frequently mentioned as a metabolic reprogramming pathway. In aerobic glycolysis, tumor cells take up excessive glucose and produce massive lactate, although there is oxygen, leading to a highly acidic tumor microenvironment (Warburg et al., 1927). Lactate has long been recognized as the “metabolic waste product”. Emerging evidence shows lactate accumulation in several neoplasms, including breast, cervical, colon and liver cancers (Jiao et al., 2018; Cheung et al., 2020). Thereafter, the excessive amounts of lactate produced will enhance acidosis within TME, thus accelerating angiogenesis, metastasis or even immunosuppression, and predicting poor survival (Vander Heiden and DeBerardinis, 2017). Therefore, lactate is the vital oncometabolite during tumor metabolic reprogramming and a promising therapeutic target.

The TME exerts a critical effect on cancer progression. Tumor-infiltrating immune cells (TIICs) in the TME have certain effect on cancer development and therapy in currently available antitumor treatments (Balkwill et al., 2012). TIICs recruited at tumor site play dual roles, they can either inhibit cancer development or promote tumor occurrence (Terlizzi et al., 2014). Activated CD8^+^ T lymphocytes can prolong survival and predict better clinical prognosis of BRCA patients (Mahmoud et al., 2011). Tumor associated macrophages (TAMs) can suppress the effect of CD8^+^ T lymphocytes and promote tumor angiogenesis and metastasis (Qian and Pollard, 2010; Noy and Pollard, 2014), causing major challenges for efficient cancer immunotherapies (Mahmoud et al., 2012). Immunotherapy, which is represented by immunological checkpoint blockade (ICB), exhibits notable therapeutic effect on some patients. However, most patients, especially those with solid tumors, experience negligible or no clinical benefit, indicating that immunotherapy is far from meeting this clinical need (Topalian et al., 2012). It is important to predict ICB response according to the characteristics of TME, so as to improve the efficacy of currently available ICB approaches and for developing novel immunotherapeutic strategies (Quail and Joyce, 2013; Ali et al., 2016). Therefore, it is important to comprehensively dissect the TME heterogeneity for identifying distinct tumor immunophenotypes and improving the prediction of immunotherapeutic responsiveness.

Tumor metabolism contributes to immunological escape. Numerous studies have demonstrated that lactate negatively affects tumor immunosurveillance by suppressing cytotoxic T lymphocytes (CTLs) in terms of their proliferation, recruitment and function (Fischer et al., 2007; Brand et al., 2016). Moreover, lactate derives from tumor can promote the polarization of macrophages to the tumor-promoting type (Colegio et al., 2014), and lactic acid pretreated bone marrow-derived mouse macrophages (BMMMs) inhibit CD8^+^ T cell growth (Ohashi et al., 2013). Murine tumors in which LDHA is inhibited produce lower levels of lactic acid; this increases the numbers of CTLs and NK cells and enhances their cytolytic activities, resulting in greater tumor inhibition when combined with PD-1 therapy than that achieved by either treatment alone (Brand et al., 2016; Daneshmandi et al., 2019). However, few comprehensive analyses have focused on the relationships between lactate production, clinical characteristics and immune cell function according to clinical data from BRCA patients. Therefore, exploration of these associations may provide insights for predicting responses to immunotherapy and understanding the mechanism underlying BRCA tumorigenesis.

The present work focused on the comprehensive evaluation of correlation of lactate levels with immune cell infiltration in BRCA, and we used a lactate score to predict patient survival and response to immunotherapy. Our work provides novel insights for improving the immunotherapeutic responses of patients, identifying diverse immune phenotypes of tumor, and promoting individualized immunotherapy.

## Methods

### Data sources

Supplementary Figure S1A displays the study flowchart. We obtained mRNA transcriptome data as well as the relevant prognostic and clinicopathological information of 1082 BRCA tumor samples and 112 normal samples in The Cancer Genome Atlas (TCGA) database (https://portal.gdc.cancer.gov/). Meanwhile, we acquired data of two eligible BRCA cohorts (GSE131769 and GSE25066) in Gene Expression Omnibus (GEO) database (https://www.ncbi.nlm.nih.gov/geo/) to conduct later analysis. We acquired raw “CELL” files, adjusted the background and normalized the quantile. Detailed information, including clinicopathological features, about these patients with BRCA were presented in Supplementary Tables S1, S2.

### Selection of potential lactate-related genes

To investigate the differences in lactate-related pathways between normal and cancer patients, the ontology enrichment scores of 12 lactate-metabolism associated pathways for each sample from TCGA-BRCA dataset were generated by “GSVA” in R package. Meanwhile, “c5.all.v7.4.symbols.gmt” gene sets obtained in the MSigDB database were adopted in gene set variation analysis (GSVA). Statistical significance was judged based on adjusted *p* < 0.05. Genes in pathways with significant differences were selected and defined as lactate-related genes (LRGs), and the ggplot2 R package was used to draw bar graphs. PCA analysis and visualization of normal and tumor samples in the TCGA-BRCA dataset based on lactate-related genes using the pca3d R package.

### Consensus clustering analysis of lactate-related genes

We utilized the “ConsensusClusterPlus” in R package to conduct consensus unsupervised clustering for classifying TCGA-BRCA patients as different lactate cluster groups based on expression levels of LRGs (Wilkerson and Hayes, 2010). The consensus matrix and cumulative distribution function (CDF) were used to calculate the optimal cluster number. To investigate the differences in biological processes among the lactate cluster subtypes, we carried out GSVA using the MSigDB database-derived hallmark gene set (h.all.v7.4.symbols.gmt).

### Assessment of tumor microenvironment immune cell infiltration and chemokine expression

We utilized CIBERSORT algorithm for evaluating 22 human immune cell subsets from each BRCA lactate subtype (Newman et al., 2015). Data about fifty-eight chemokines were obtained from the MSigDB database for comparing differential expression among lactate clusters by using ggplot2 and ggpubr R packages.

### Association of lactate subtypes with breast cancer clinical characteristics as well as prognostic outcome

For examining whether the consensus clustering-identified lactate subtypes were of clinical significance, this work compared correlation among clinicopathological features, molecular subtypes, as well as prognostic outcome. Typically, patient features were stage, PAM50 subtype, TNM stage, and immune subtype. We used single-sample GSEA (ssGSEA) to quantify the difference of clinical feature for each patient in different clusters using the “GSVA” package in R. By adopting “survminer” and “survival” in R package, we plotted Kaplan–Meier (KM) curves to compare recurrence-free survival (RFS) across diverse subtypes.

### Differentially expressed genes identification and functional enrichment

This work utilized “limma” in R package to identify DEGs among diverse lactate clusters, and the cutoffs were adjusted *p* < 0.05 and fold-change (FC) > 1.5 (Ritchie et al., 2015). For better exploring lactate cluster-associated DEGs’ possible effects and identifying relevant gene functions as well as pathways, this work conducted functional enrichment of the DEGs using “clusterprofler” package.

### Construction and validation of the lactate score

This work determined a lactate score for quantifying lactate metabolism-patterns in different tumor samples. Firstly, after clustering of lactate subtypes, DEGs identified from the different lactate clusters were extracted. Secondly, all patients were divided as three gene clusters (lactate gene cluster A, B, C) in further unsupervised clustering analysis to analyze overlapping DEGs. We defined gene cluster number and stability using the consensus clustering algorithm. Thereafter, prognosis of all genes incorporated into the model was analyzed by univariate Cox regression. Thereafter, this work selected significant prognostic genes in subsequent analyses. Further, lactate gene signatures were constructed by principal component analysis (PCA), with principal components 1/2 (PC1/PC2) being chosen to be signature scores. Later, this study determined lactate score by gene expression grade index (GGI) (Sotiriou et al., 2006; Zeng et al., 2019):

$$Lactate\;score=\sum \left(PC1_{i}\;+\;PC2_{i}\right)$$

in the formula, i stands for LRG expression levels. Patients were classified as low- or high-risk group according to lactate score using the survminer R package. We plotted KM survival curves and receiver operating characteristic (ROC) curves with ‘survival ROC’ in R package to predict survival based on the lactate score. The area under the curve (AUC) was calculated for assessing lactate score’s prognosis prediction accuracy (Yu et al., 2012). Besides, this work further validated the role of the lactate score algorithm in two independent BRCA validation sets, namely, GSE131769 and GSE25066.

### Nomogram construction and validation

This study constructed a prognosis prediction nomogram by incorporating risk score and clinical features with “rms” package in line with prognosis analytic results. All variables in nomogram were assigned with corresponding scores, which were later added to obtain the overall score. This work also plotted 1-year time-dependent ROC (t-ROC) curves for nomogram assessment. Besides, we depicted the nomogram prediction ability based on calibration plots by comparing the 1-, 2-, and 3-year survival rates predicted with those measured values.

### Immunotherapeutic and drug susceptibility analysis

The present work collected patient information in two prospective clinical studies conducted among patients with advanced clear cell renal cell carcinoma (NSCLC)–CheckMate 010 (CM-010; NCT01354431) (Motzer et al., 2015) together with CheckMate 025 (CM-025; NCT01668784) (Motzer et al., 2018) using anti-PD-1 antibody (nivolumab). We used the web platform TIDE (http://tide.dfci.harvard.edu/) for predicting ICB therapy responses (Jiang et al., 2018). Meanwhile, we downloaded RNA-seq data in CheckMate 010 and 025 from Supplementary Table S4 of PMID: 32472114 (Braun et al., 2020). The “pRRophetic” package was used for exploring different efficacy achieved by chemotherapeutics among 2 patient groups. For chemotherapeutics frequently utilized for BRCA treatment, their semi-inhibitory concentrations (IC50) were determined.

### Statistical analyses

Differences across diverse groups were compared by Kruskal–Wallis test and one-way ANOVA. R version 4.1.0 was employed for statistical analysis. *p* < 0.05 stood for significant difference.

## Results

### Lactate-related genes screening in breast cancer

Supplementary Figure S1 displays the analytical process in this work. A total of 12 lactate metabolism-related pathways were screened and found to be enriched in BRCA tissues compared with normal tissues (Supplementary Figure S2A). The number of genes associated with each pathway and the different *p* value were shown in Supplementary Figure S2B. A total of 204 genes associated with the top 3 enriched pathways that had the smallest *p* values were considered LRGs (Supplementary Table S3). Based on the expression level of these 204 genes, BRCA samples can be distinguished from normal samples using PCA (Supplementary Figure S2C).

### Genetic and transcriptional alterations of lactate-related genes in breast cancer

**FIGURE 1:**
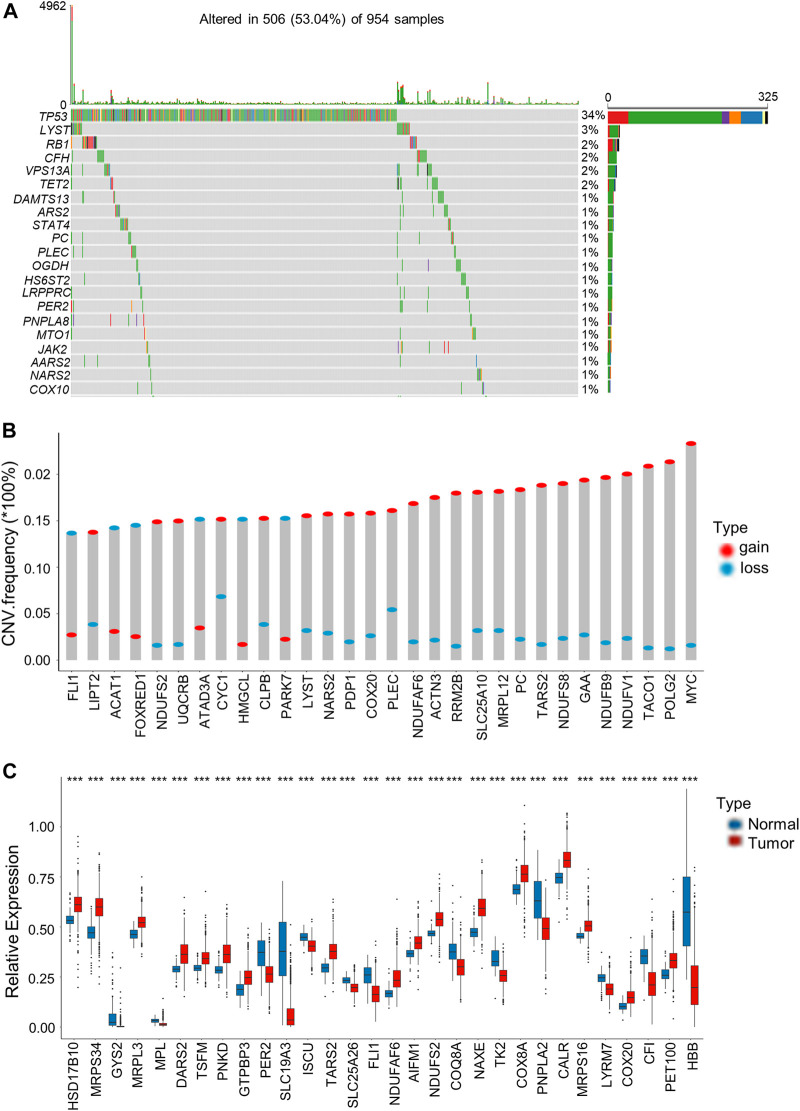
Genetic and transcriptional alterations of LRGs in BRCA. **(A)** The top 30 LRGs with the highest mutation frequency in 506 patients with BRCA based on TCGA-BRCA cohort. The columns represent diverse patients. The top bar plot presents the TMB. The number on the right stand for gene mutation frequency. Bar plot on the right presents variant proportions. **(B)** Those top 30 LRGs with the most significant frequencies of CNV gain and loss. The column height stands for alteration frequency. Red and blue dots stand for amplification and deletion frequencies, separately. **(C)** Those top 30 differentially expressed LRGs between BRCA and normal samples. Asterisks represent the *p*-values (***p < 0.001).

We then analyzed somatic mutations and copy number variations (CNVs) of LRGs in BRCA samples. Among the 954 BRCA samples, 506 (53.04%) had mutations in the LRGs. Among these genes, *TP53* had the highest mutation frequency (34%), followed by *LYST* (Figure 1A). Afterwards, somatic copy number alterations of the above LRGs were analyzed, as a result, CNVs were commonly seen within the LRGs; most of these CNV alterations represented the amplification in copy number (Figure 1B). We also analyzed mRNA expression in BRCA samples compared with normal tissues, as a result, many LRGs showed positive relation to CNVs. For LRGs that harbored CNV gain, including *NDUFAF6*, *COX20*, *TARS2*, their levels markedly elevated within BRCA tissues compared to healthy controls, whereas LRGs with CNV loss, such as *FLI1*, were expressed at lower levels in BRCA samples (Figure 1C).

### Identification of lactate subtypes and biological characteristics in breast cancer

**FIGURE 2:**
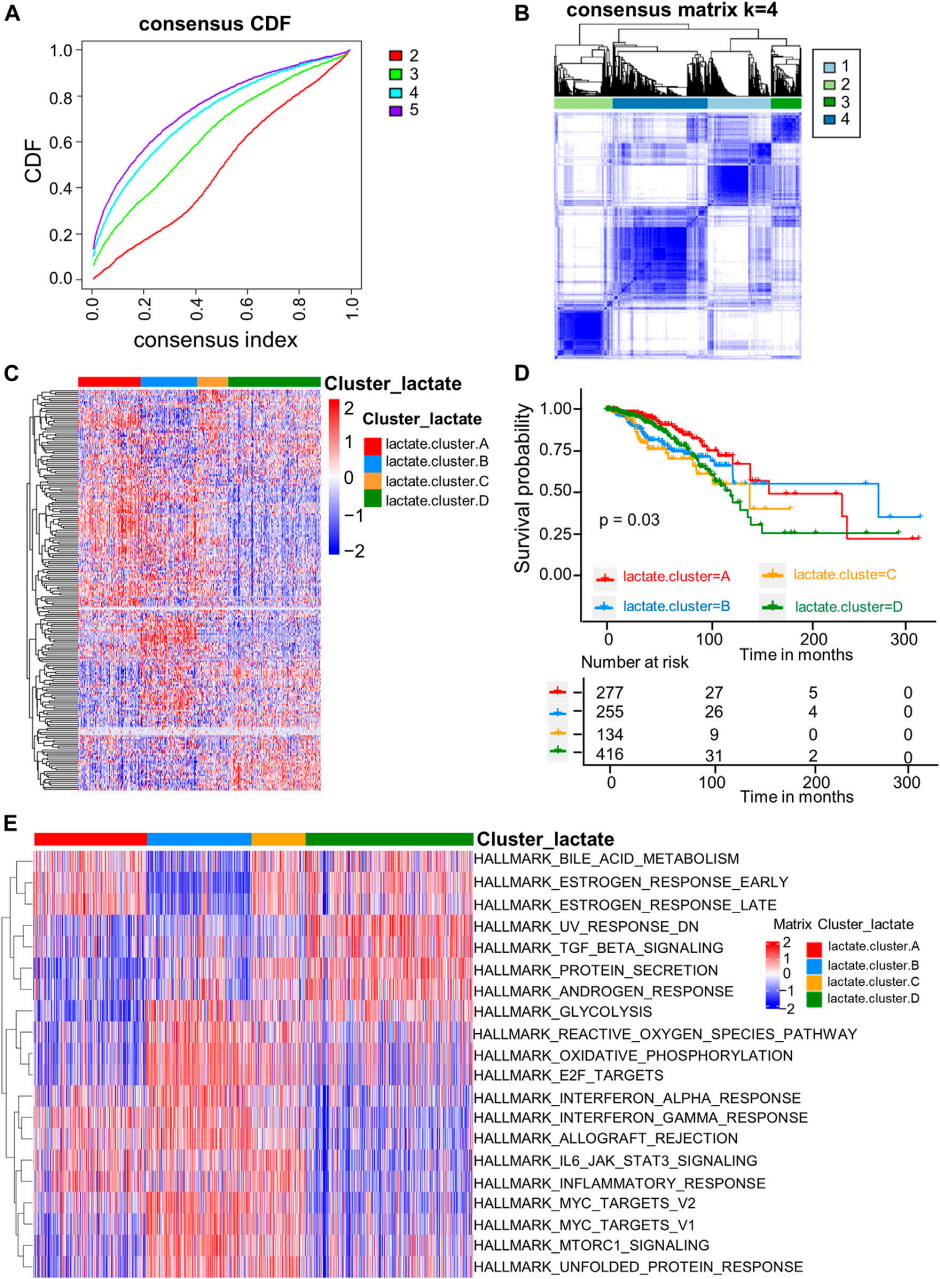
LRG subtypes as well as biological features among four different subtypes classified through consensus clustering. **(A)** Cumulative distribution function (CDF) of consensus clustering at k = 2–5. **(B)** Consensus matrix heatmap that defined four clusters (k = 4) as well as the relation area. **(C)** Heatmaps of the hierarchical clustering of differential gene expression in the four clusters. **(D)** Survival analyses of the four lactate subtypes. KM curves (*p*-value = 0.03, log-rank) showed that survival was different among four clusters. **(E)** GSVA of 50 hallmark gene sets among the four clusters.

For better exploring LRG expression features within BRCA, we conducted consensus clustering analysis for categorizing BRCA patients according to the expression levels of the 204 LRGs (Figure 2A). As a result, k = 4 was optimal to sort the whole cohort as lactate clusters A-D (Figure 2B). The four distinct clusters showed differences in the expression of LRGs (Figure 2C). The Kaplan–Meier curves showed that patients in lactate cluster D showed the worst RFS, and those of lactate cluster A had better RFS (*p* = 0.03 upon log-rank test; Figure 2D). For exploring biological behaviors among the different lactate clusters, GSVA of hallmark gene sets in MSigDB were carried out. Lactate clusters A and B were enriched in immune activation-related pathways, such as the inflammatory response, interferon alpha response, and interferon gamma response (Figure 2E). Lactate clusters D were enriched with the TGF beta signaling pathway (Figure 2E). These results suggested that the lactate cluster subtype may be associated with different immune response in the TME.

### Characteristics of infiltrating immune cells in the tumor microenvironments of distinct lactate subtypes

**FIGURE 3:**
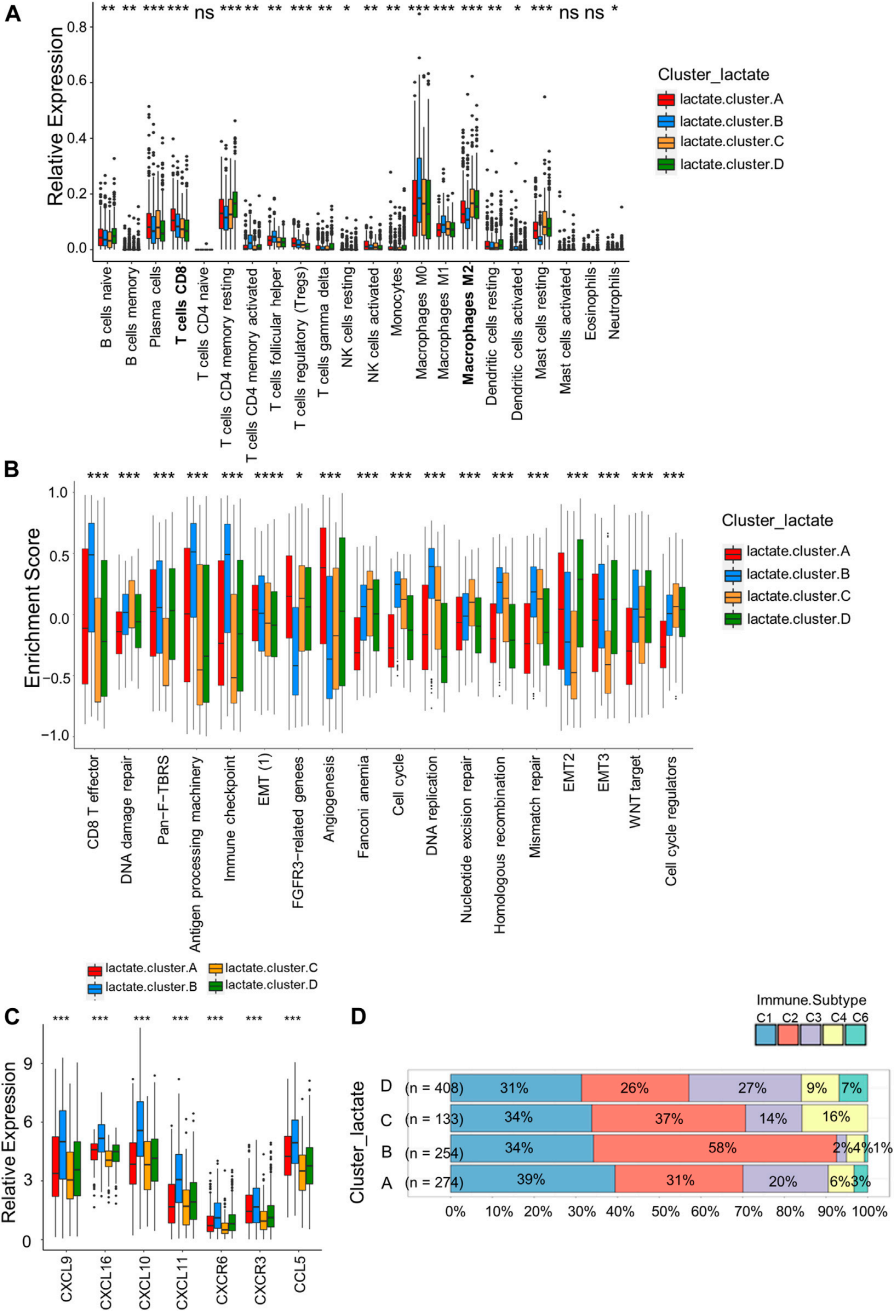
Correlations of immune cells infiltrations into the TME of the four lactate subtypes. **(A)** Enrichment levels of 22 infiltrating immune cells among four lactate subtypes. **(B)** GSVA enrichment scores of the classical gene signatures among the 4 lactate clusters using the ESTIMATE package. **(C)** Expressions of T cell-associated chemokines in the four lactate subtypes. **(D)** Correlation between immune subtypes abundances and the four lactate clusters. Differences among 3 gene clusters were compared by chi-square test (*p* < 0.001). Asterisks represent *p*-values (*p < 0.05; **p < 0.01; ***p < 0.001).

For investigating LRGs’ effect on TME in BRCA, we analyzed the correlations between the four subtypes and the numbers of 22 immune cell subsets in diverse BRCA samples by adopting the CIBERSORT algorithm. Lactate B was markedly enriched in CD8^+^T cells, M0 and M1 macrophages, whereas M2 macrophages showed an increased level within lactate clusters C and D (Figure 3A). Similarly, the CD8^+^T effector and antigen processing machinery gene signatures increased within lactate clusters B (Figure 3B). We also observed enrichment of immune checkpoint genes in cluster B (Figure 3B). Moreover, we analyzed the expression of 57 chemokines among the four subtypes (Supplementary Table S6). T cell-recruiting chemokines, including *CXCL9*, *CXCL10* and *CXCL11*, were higher in lactate clusters B, which was consistent with the higher T cell infiltration (Figure 3C). A landscape of the tumor immune microenvironment derived from immunogenomics data classified BRCA into 5 immune subtypes: C1 (wound healing), C2 (IFN-g dominant), C3 (inflammatory), C4 (lymphocyte depleted) and C6 (TGF-b dominant) (Thorsson et al., 2018). Lactate clusters C and D had higher proportions of the C4 and C6 subtypes compared to lactate clusters A and B (Figure 3D). BRCA subtypes C4 and C6 are reported to be associated with a worse prognosis (Thorsson et al., 2019). Collectively, clusters B were classified as having the immune-inflammatory phenotype, which had the feature of immune activation. Lactate clusters C and D were divided into immune-desert phenotypes, which had the feature of immunosuppression.

### Identification of lactate gene subtypes and clinical characteristics

**FIGURE 4:**
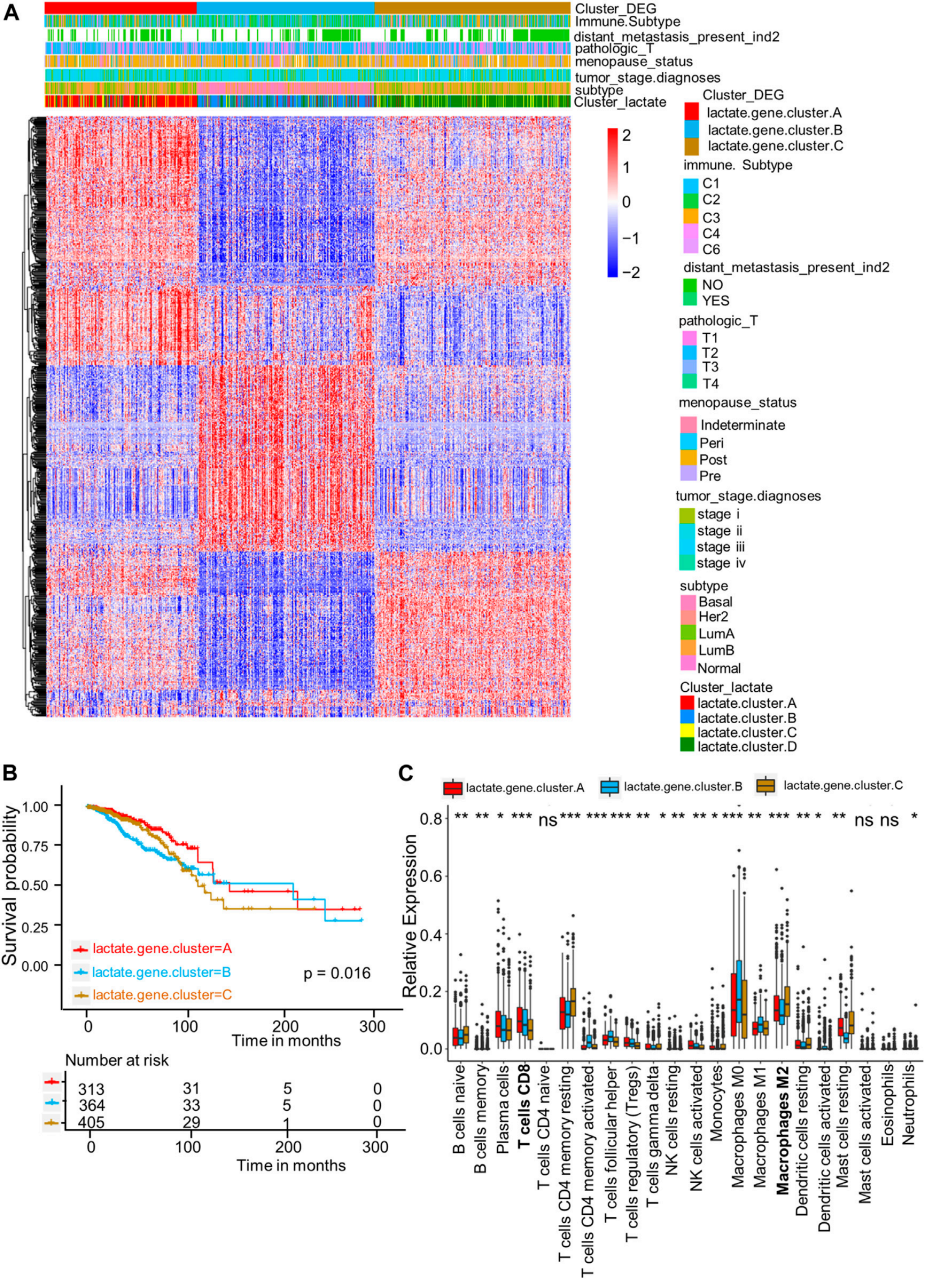
DEGs-based gene subtype identification. **(A)** Relationships among clinicopathologic features, lactate clusters, and the three gene subtypes. **(B)** KM analysis for RFS among three gene subtypes (*p* = 0.016, log-rank tests). **(C)** Enrichment levels of 22 infiltrating immune cells among three gene subtypes. Asterisks represent *p*-values (*p < 0.05; **p < 0.01; ***p < 0.001).

For better exploring diverse lactate subtypes’ possible biological behaviors, 717 DEGs related to lactate subtype were discovered by limma package (Supplementary Table S4). We then used the consensus clustering analysis to divide patients as 3 genomic subtypes according to the 717 DEGs, including lactate gene clusters A–C (Figure 4A). In addition, as revealed by KM curves, patients of lactate gene cluster C showed the worst RFS, while those of lactate gene cluster A had prolonged RFS (Figure 4B). We also used the CIBERSORT algorithm for assessing the relation of lactate gene clusters with immune cells abundance within TME. Consistent with the results of lactate cluster analysis, the infiltration of M0 and M1 macrophages, as well as CD8^+^ T cells remarkably increased in lactate gene cluster B than in lactate gene cluster C, while lactate gene cluster C was markedly enriched in M2 macrophages (Figure 4C).

### Lactate score establishment and clinicopathological feature evaluation

**FIGURE 5:**
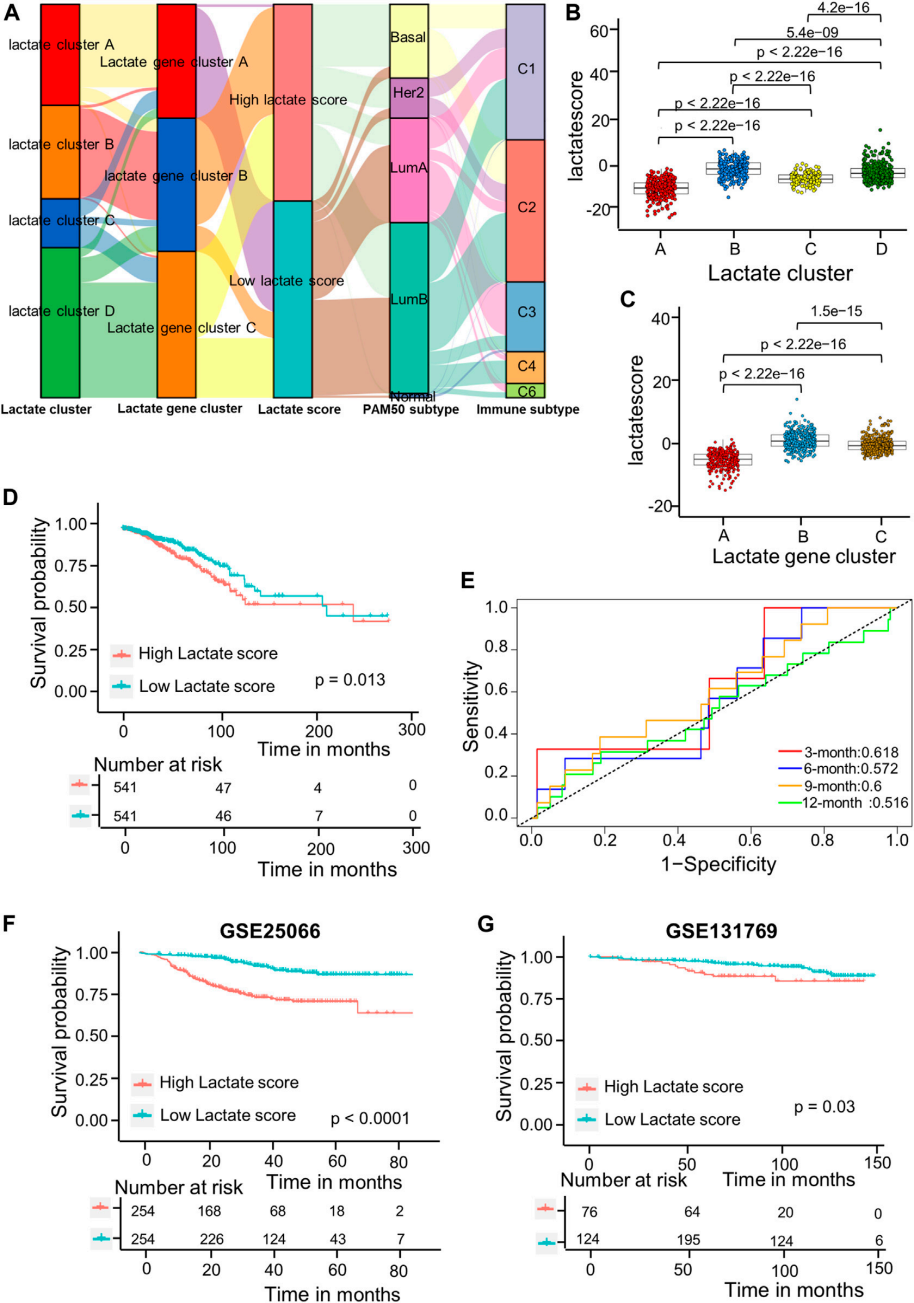
Lactate score construction. **(A)** Alluvial diagram showing the association of the lactate cluster, lactate gene cluster, lactate score, PAM50 subtype and immune subtype. **(B)** Differences in lactate score among the four lactate clusters based on TCGA-BRCA cohort. Differences among those 3 gene clusters were compared by Kruskal–Wallis test (*p* < 0.001). **(C)** Differences of lactate scores among those 3 gene clusters based on TCGA-BRCA cohort (*p* < 0.001, Kruskal–Wallis test). **(D)** Kaplan-Meier analysis of the RFS in high versus low lactate score patients. **(E)** ROC curves to predict sensitivity and specificity of the lactate score in predicting 3-, 6-, 9-, and 12-month survival. **(F)** KM analysis for RFS of both groups in the GSE25066 cohort. **(G)** KM analysis of the RFS between the two groups in GSE131769 cohort. ROC, receiver operating characteristic; RFS, recurrence-free survival.

As shown in the above results, patients in cluster B have high immune infiltration but lower survival. Therefore, we wanted to establish a simpler lactate score based on lactate subtype-related DEGs for prognosis of BRCA. Univariate Cox regression analysis was carried out for identifying 717 LRGs for their prognostic significance. As a result, 79 RFS-related genes were identified (*p* < 0 0.05); these genes were used to generate the lactate score by using PCA algorithms (Supplementary Table S5). Associations of lactate cluster, lactate gene cluster, lactate score, PAM50 subtype and immune subtype are displayed in the alluvial diagram (Figure 5A). Most patients in clusters C and D had higher lactate scores and were classified into aggressive basal and her2 subtypes (Figure 5A). The Kruskal-Wallis test revealed that lactate cluster A had smallest median score, whereas lactate cluster B showed the greatest score (Figure 5B). There was higher CD8 T cell score in lactate cluster B, but the expressions of immune checkpoint-related molecules are also high (Figure 3B). However, the survival of lactate cluster B patients within 100 months are worse than cluster A, suggesting that there are other factors such as T cell exhaustion and high myeloid cell infiltration that affect tumor progression. More importantly, lactate gene cluster B had significantly increased lactate score in comparison with others, and lactate gene cluster A showed lowest score (Figure 5C). According to KM curves, patients who had decreased lactate scores were associated with markedly more favorable overall survival (OS) compared with those having increased lactate scores (*p* = 0.013 upon log-rank test; Figure 5D). Additionally, AUC values of lactate risk score in predicting the 3-, 6-, 9-, and 12-month survival rates were 0.618, 0.572, 0.6, and 0.516, separately (Figure 5E). For validating lactate score’s prognostic value, this work determined lactate scores based on one internal (training set) as well as two external validation cohorts (GSE25066 and GSE131769). We classified the patients as high- or low-risk group based on formula utilized in training cohort. As suggested by survival analysis, low-risk patients had markedly superior survival to high-risk patients (*p* = 0.03 upon log-rank; Figures 5F,G).

**FIGURE 6:**
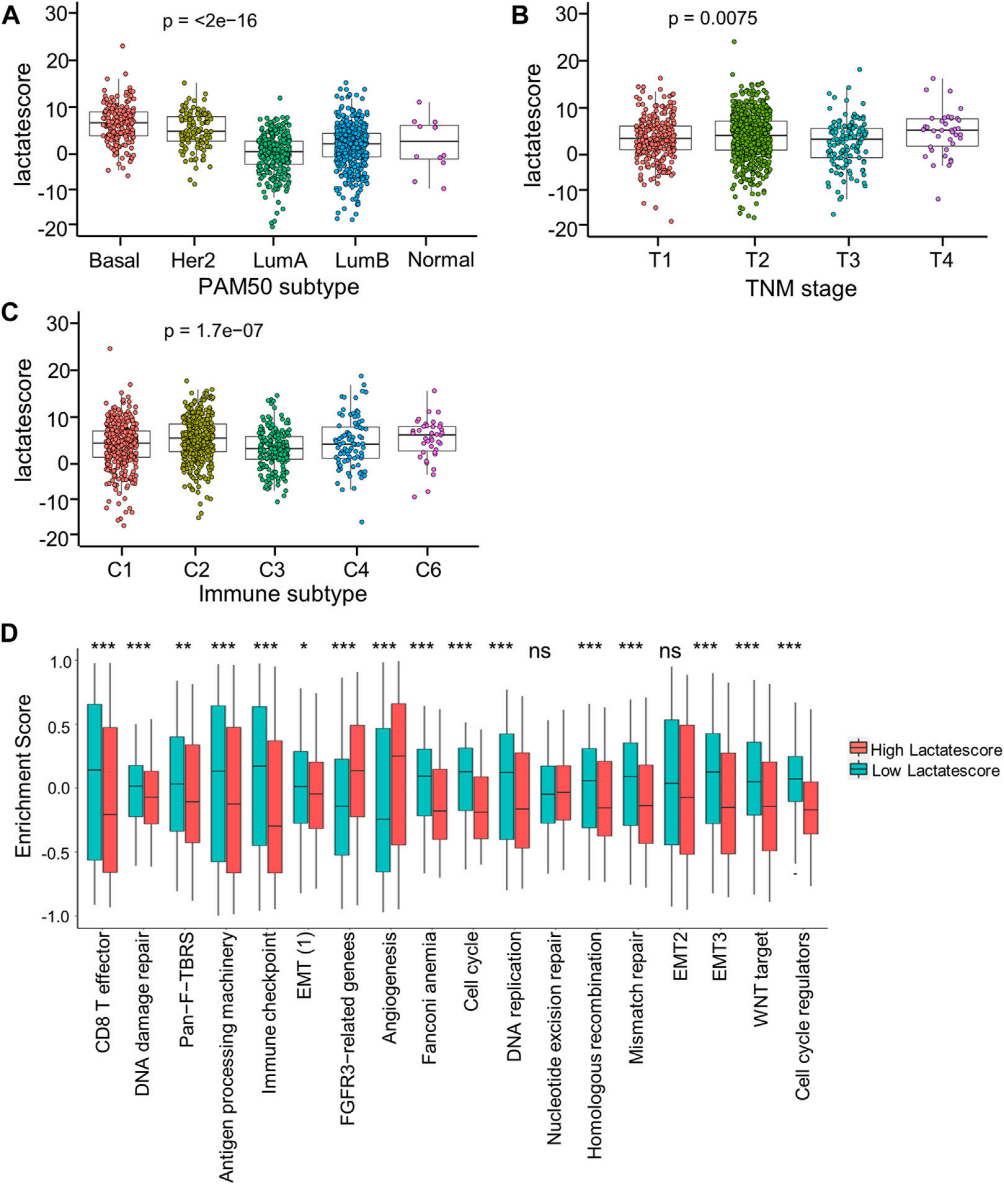
Correlation between lactate score and clinical characteristics. **(A)** Lactate score of different PAM50 molecular subtypes. **(B)** Lactate score in subtypes of different TNM stages. **(C)** Lactate score of different immune subtypes. **(D)** GSVA enrichment scores of the classical gene signatures of high-versus low-score patients by ESTIMATE algorithm. Asterisks represent *p*-values (*p < 0.05; **p < 0.01; ***p < 0.001).

For investigating lactate score’s role in clinical features, this work analyzed correlations of the lactate score with diverse clinical characteristic (PAM50 subtype, TNM stage, and immune subtype). Patients in the Basal and Her2 subgroups had markedly increased lactate scores, which were more aggressive tumors (Figure 6A). Moreover, the stage IV subgroup had the highest lactate scores compared with the other stage groups (Figure 6B). Consistent with the immune subtype analysis in the lactate clusters, the C6 immune subtypes had higher lactate scores than the C1and C3 subtypes (Figure 6C). Similarly, the analyses of the immune pathway activities suggested that low lactate scores were markedly related to CD8^+^ T effector and antigen processing machinery gene signatures, while angiogenesis gene signatures were significantly enhanced in high lactate scores (Figure 6D).

Based on these findings, a low lactate score was markedly related to immune-activation, whereas a high lactate score was related to stromal-activation. Moreover, lactate score performed well in assessing lactate metabolism profiles among different tumors, which also assessed immune cell in TME.

### Development of a nomogram based on the lactate score to predict survival

**FIGURE 7:**
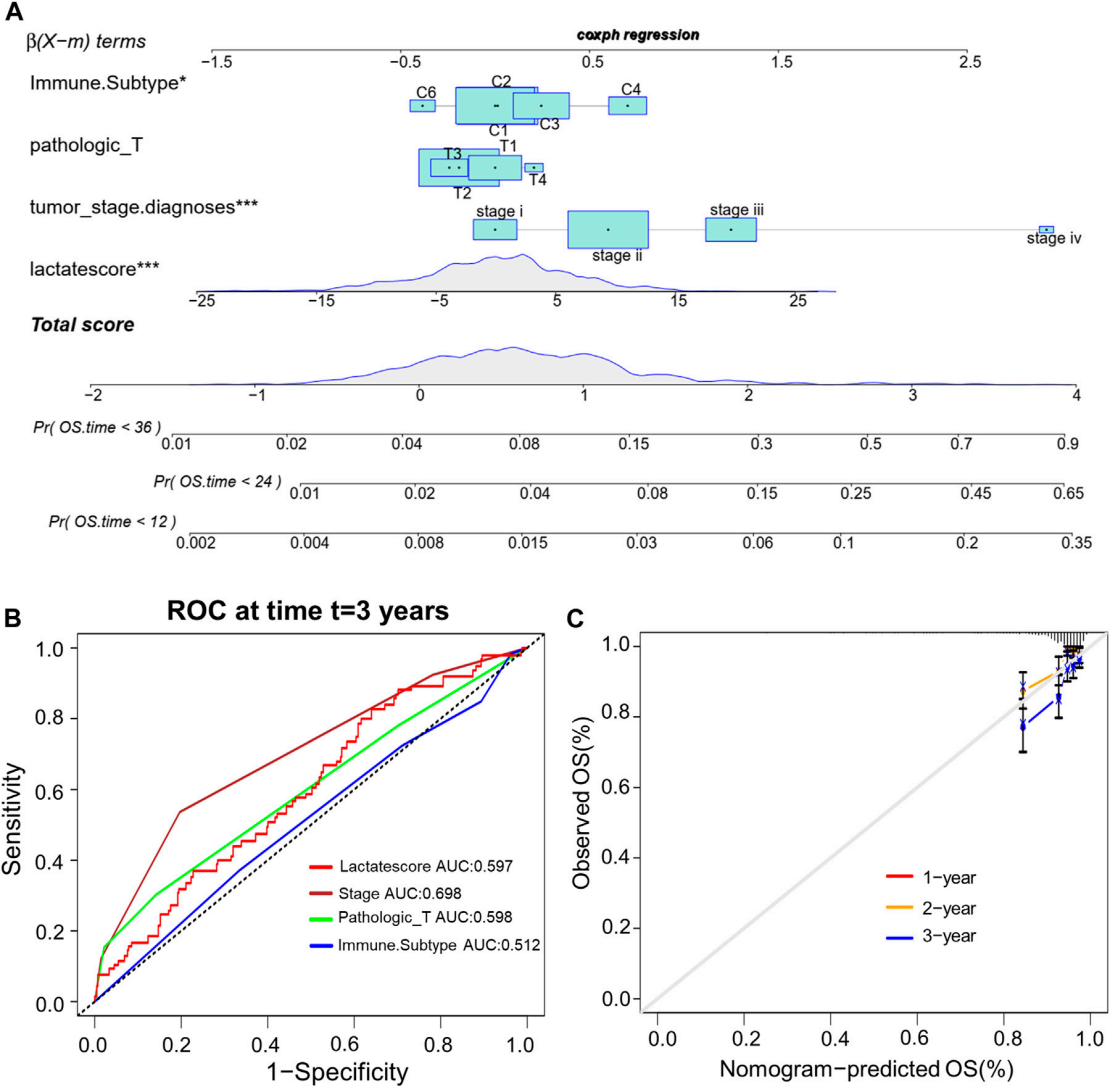
Establishment of the comprehensive nomogram based on TCGA-BRCA cohort. **(A)** Nomogram development according to immune subtype, pathologic_T, tumor_stage.diagnoses and lactate score. **(B)** ROC curves for our constructed nomogram. **(C)** Calibration plots for our constructed nomogram.

This work analyzed the feasibility of lactate score to independently predict the prognosis of BRCA. As revealed by multivariate regression including patient’ immune subtype, pathologic_T, tumor_stage and lactate score, lactate score was the vigorous factor independently predicted patient prognosis [HR 0.6 (0.42–0.85); Supplementary Figure S3]. Then, we established a comprehensive nomogram that integrated the lactate score with three other clinicopathological factors for predicting RFS at 1-, 2 and 3-year (Figure 7A). The nomogram-predicted value was compared with those predicted by stage, pathologic_T and immune subtype, and the 3-year AUCs were 0.597, 0.698, 0.598 and 0.512 separately, suggesting that our nomogram exhibited superior ability to predict survival compared to the immune subtype (Figure 7B). According to the subsequent calibration plots, our nomogram achieved similar performance to the optimal model (Figure 7C).

### Lactate score in the prediction of response to therapies

**FIGURE 8:**
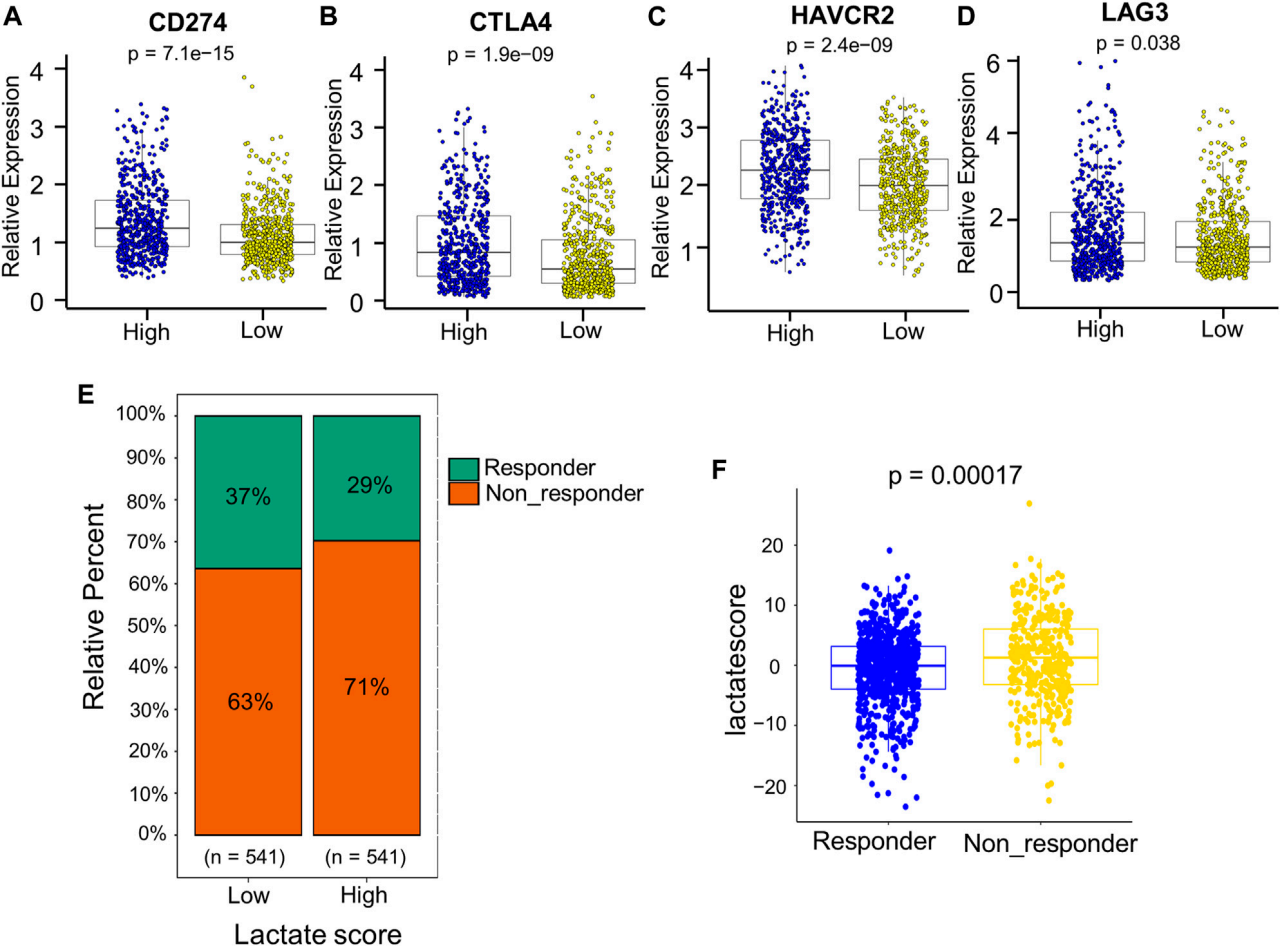
Comprehensive analysis of the role of the lactate score in therapy. **(A–D)** Expression of inhibitory immune checkpoints, including *PD-L1* (*CD274*), *CTLA4*, *TIM3* (*HAVCR2*) and *LAG3*, of low- and high-risk patients. **(E)** Percentage of patients responding to PD-1 blockade immunotherapy of low or high lactate score groups. **(F)** Distribution of lactate scores in distinct anti-PD-1 clinical response groups.

Patients with an increased tumor mutation burden (TMB) show durable clinical responses to immunotherapy (Snyder et al., 2014). Thereafter, this work examined the different somatic mutation distributions of high and low lactate score patients from TCGA-BRCA cohort. According to Supplementary Figure S4, the TMB was comparable between high and low lactate score groups. Patients who had increased lactate scores were associated with increased *TP53* and *XIRP2* mutation frequencies compared with low lactate score ones. However, *PIK3CA* and *TTN* mutation frequencies showed the exact opposite results (Supplementary Figure S4). Additionally, the expression of inhibitory immune checkpoints, including *CTLA4*, *PD-L1* (*CD274*), *TIM3* (*HAVCR2*) and *LAG3*, were higher in the high lactate score group (Figures 8A–D). Other immune checkpoint molecules involved in T cell activation were expressed at higher levels for patients having low lactate scores (Supplementary Figure S5).Therefore, these findings suggest that the differential expression of LRGs in tumors is a potentially important factor that affects immunotherapeutic responses. This work further found that the low lactate score group had significantly more treatment responders in the anti-PD-1 cohort (CheckMate 010; NCT01354431 and CheckMate 025; NCT01668784, Figure 8E). Similarly, the nonresponders had a significantly increased lactate score compared to the responders (Figure 8F). Subsequently, chemotherapeutics utilized to treat BRCA at present were chosen for evaluating their sensitivities in high and low lactate score patients. Interestingly, the IC50 values for chemotherapeutics, such as paclitaxel, docetaxel, doxorubicin and gemcitabine, decreased among low lactate score patients, compared with those in the high lactate score group (Supplementary Figure S6). Collectively, lactate score was associated with therapy responses.

## Discussion

Lactate has an indispensable effect on establishing an immunosuppressive microenvironment, which favors tumor progression (Gabrilovich et al., 2012; Morrot et al., 2018). However, many studies mainly focus on one immune cell type or one lactate metabolism-related gene, and the combined effects of multiple LRGs on immune infiltration into the TME are not comprehensively elucidated. Consequently, determining the role of different lactate metabolism profiles in immune cell infiltration into the TME will help to understand the mechanisms underlying BRCA tumorigenesis, and develop more effective immunotherapy strategies.

This work illustrated the global alterations of LRGs within BRCA at the genetic and transcriptional levels. Four different molecular subtypes were discovered according to 204 LRGs. These four patterns had significantly distinct TME cell infiltration characteristics. Lactate clusters A and B were associated with the feature of adaptive immunity activation, which was associated with an immune-inflammatory phenotype, also known as a hot tumor with the infiltration of massive activated immune cell (Chen and Mellman, 2017). Lactate clusters C and D had the feature of immunosuppression, associated with immune-desert phenotype that display immune tolerance, with little or no activated T cells (Joyce and Fearon, 2015). Conforming to these definitions, this work discovered that lactate clusters A and B were markedly enriched in CD8^+^ T cells, antigen processing machinery gene signatures and chemokines for T cell–recruiting, like *CXCL9*-*CXCL11*.

Furthermore, DEGs among the four lactate subtypes were considered lactate-related signature genes. We identified three gene subtypes based on these DEGs, and they were markedly related to immune as well as stromal activation. As a result, LRGs are important for evaluating the responses to immunotherapy and clinical outcomes of BRCA. Therefore, a scoring system were established for evaluating lactate metabolism patterns in different BRCA patients. Lactate subtype with the immune-desert phenotype had an increased lactate score, while subtype with the immune-inflamed phenotype showed the decreased lactate score. Clinicopathological features, prognoses, TMEs, mutations, immune checkpoints expression levels, immunotherapy and drug susceptibilities were different between patients who had high and low lactate scores. Correspondingly, lactate score, immune subtype, pathologic_T, and tumor stage, were integrated to construct the comprehensive nomogram, for the sake of improving the use and accuracy of lactate score. Our integrated nomogram is utilized to stratify prognosis of BRCA patients, which helps to better understand the mechanism underlying BRCA tumorigenesis and provides new important insights for the development of immunotherapy.

Immunotherapy brings new hopes for treating cancers, in particular for lymphoma, melanoma, renal cell cancer, and non-small-cell lung cancer (NSCLC) (Waldman et al., 2020). However, patients with BRCA show limited clinical benefits after immunotherapy due to the heterogeneity of the TME in BRCA tumorigenesis and progression (Emens, 2018). The TME facilitates immunosuppression and limits anticancer immune responses. TME has the feature of recruiting suppressive immune cells, physical barriers to immune infiltration, and upregulated immunosuppressive ligand expression on tumor cells (Turley et al., 2015). Lactate derived from tumor can regulate immune cell function, thereby facilitating to establish the immunosuppressive TME favoring impaired efficacy of immunotherapy (Feichtinger and Lang, 2019). In this study, M1 macrophages and CD8^+^ T cells were significantly enriched into low lactate score group, which predicted the good prognostic outcome, and high lactate score group showed a higher enrichment level of M2 macrophages, which predicted poor prognostic outcome. Immunosuppressive M2 macrophages, are barriers to cancer immunotherapy and enhance the metastasis of BRCA (Qiu et al., 2018; Larionova et al., 2020). Immune checkpoints inhibit antitumor immune response within TME (Nishino et al., 2017). Accordingly, high lactate score group showed higher inhibitory immune checkpoints levels, like *CTLA4*, *PD-L1* (*CD274*), or *TIM3* (*HAVCR2*). Hence, antitumor effect on patients showing increased high lactate scores is possibly inhibited *via* the excessive amounts of M2 macrophages together with the overexpression of inhibitory immune checkpoints. This work verified that lactate score was effective on predicting patient’ responses to anti-PD-1 immunotherapy.

Certain limitations should be noted in this work. Firstly, experimental data for evaluating the biological behavior-related mechanisms are lacking. Secondly, more multi-center studies with large sample size should be conducted for validating the above results.

We developed a novel lactate score based on LRGs, and this score could comprehensively evaluate TME immune cell infiltration and prognosis for BRCA patients. Besides, lactate score was utilized for assessing patient clinical features and anti-PD-1 immunotherapeutic responses. Our work suggested that targeting LRGs could turn the relatively “cold tumors” to the “hot tumors”, which help to develop the new immunotherapeutic agents and new drug combination strategies.

## Data Availability

The datasets presented in this study can be found in online repositories. The names of the repository/repositories and accession number(s) can be found in the article/Supplementary Material.
