# Supplementary material for: Lactate score predicts survival, immune cell infiltration and response to immunotherapy in breast cancer
Source: Front Genet. 2022 Aug 15;13:943849. doi: 10.3389/fgene.2022.943849 (PMC9421043; doi:10.3389/fgene.2022.943849)
Supplement: Supplementary file 7 [file DataSheet1.DOCX]

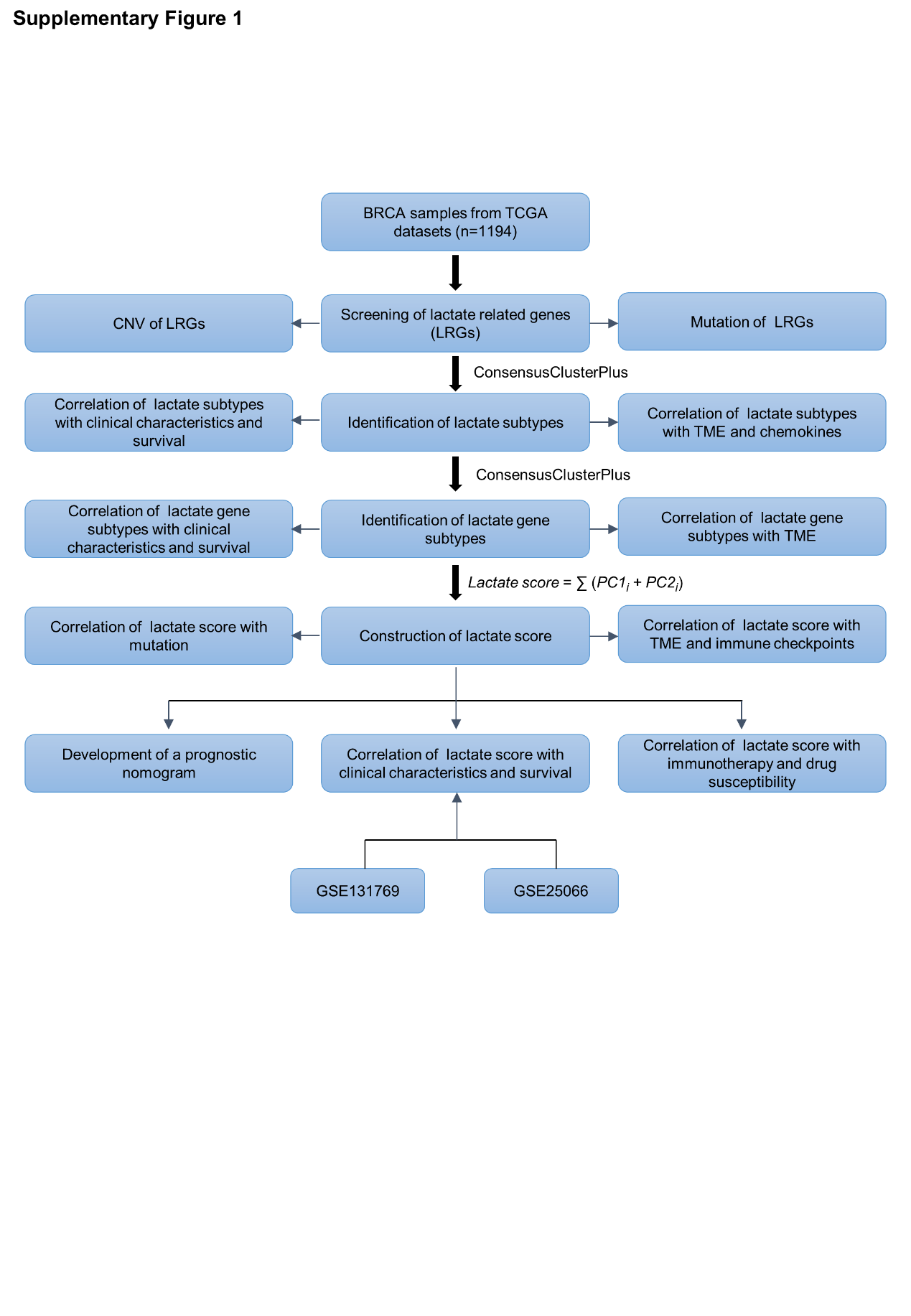


**Supplementary Figure 1.** The workflow of our study.


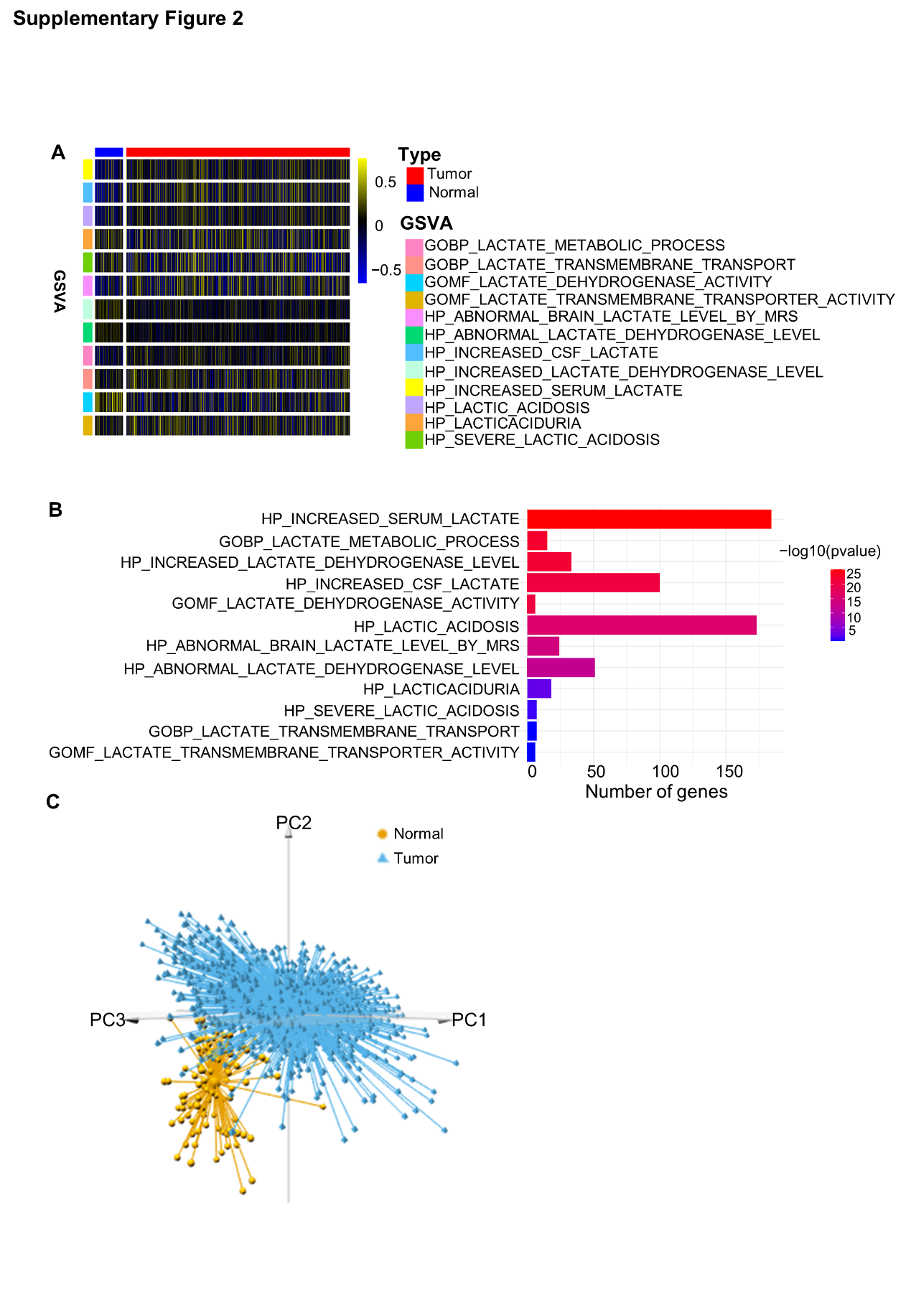


**Supplementary Figure 2.** Identification of lactate-related genes. **(A)** GSVA enrichment analysis showing the lactate-related pathways in BRCA and normal tissues. **(B)** GO enrichment analyses of 12 lactate-related pathways among tumor tissues and normal tissues. **(C)** PCA of the expression profiles of 204 lactate related genes to distinguish tumor samples from normal samples in the TCGA-BRCA cohort. Tumors are labeled blue, and normal samples are labeled yellow.


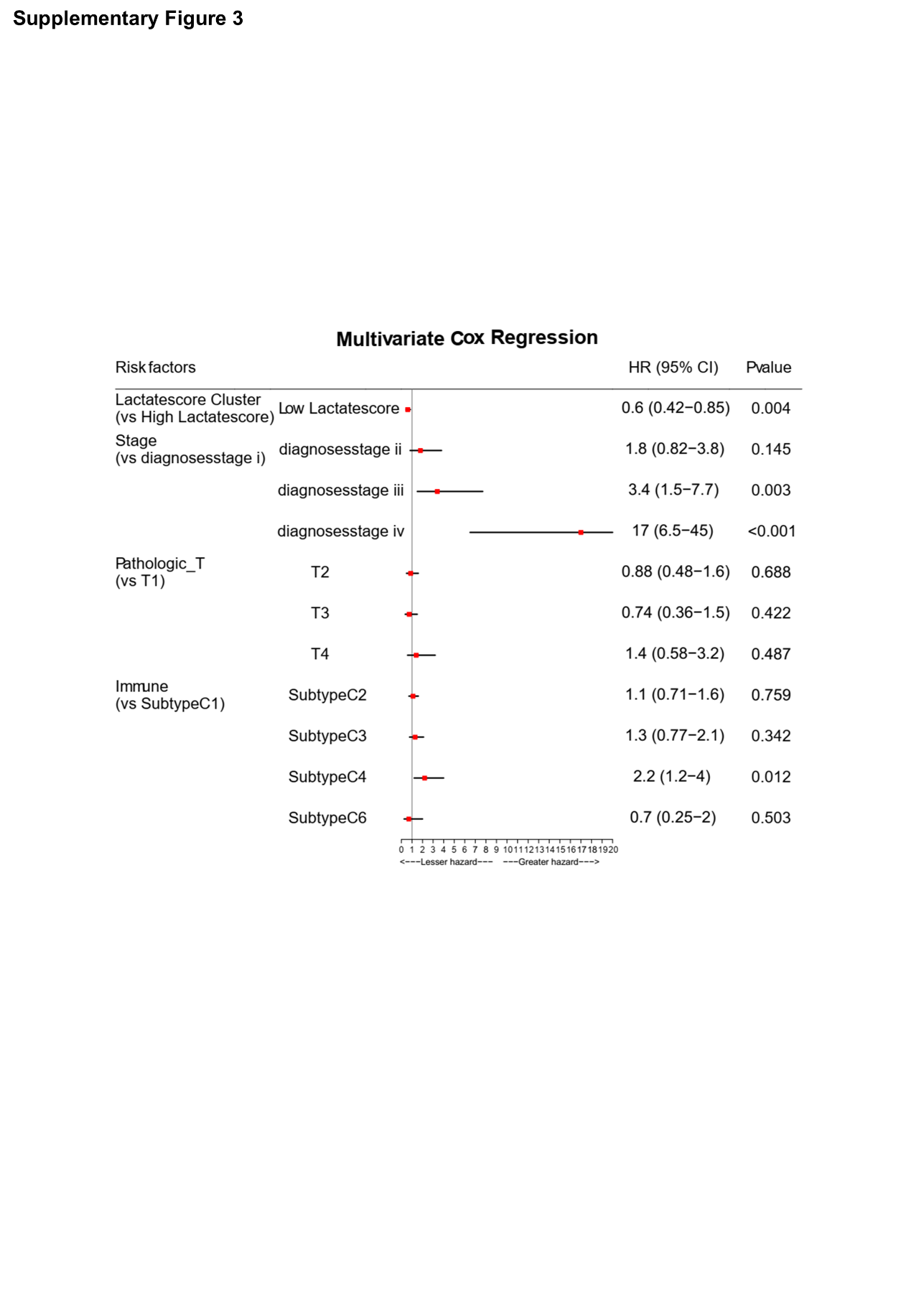


**Supplementary Figure 3.** Univariate and multivariate analyses showed the prognostic value of the lactate score.


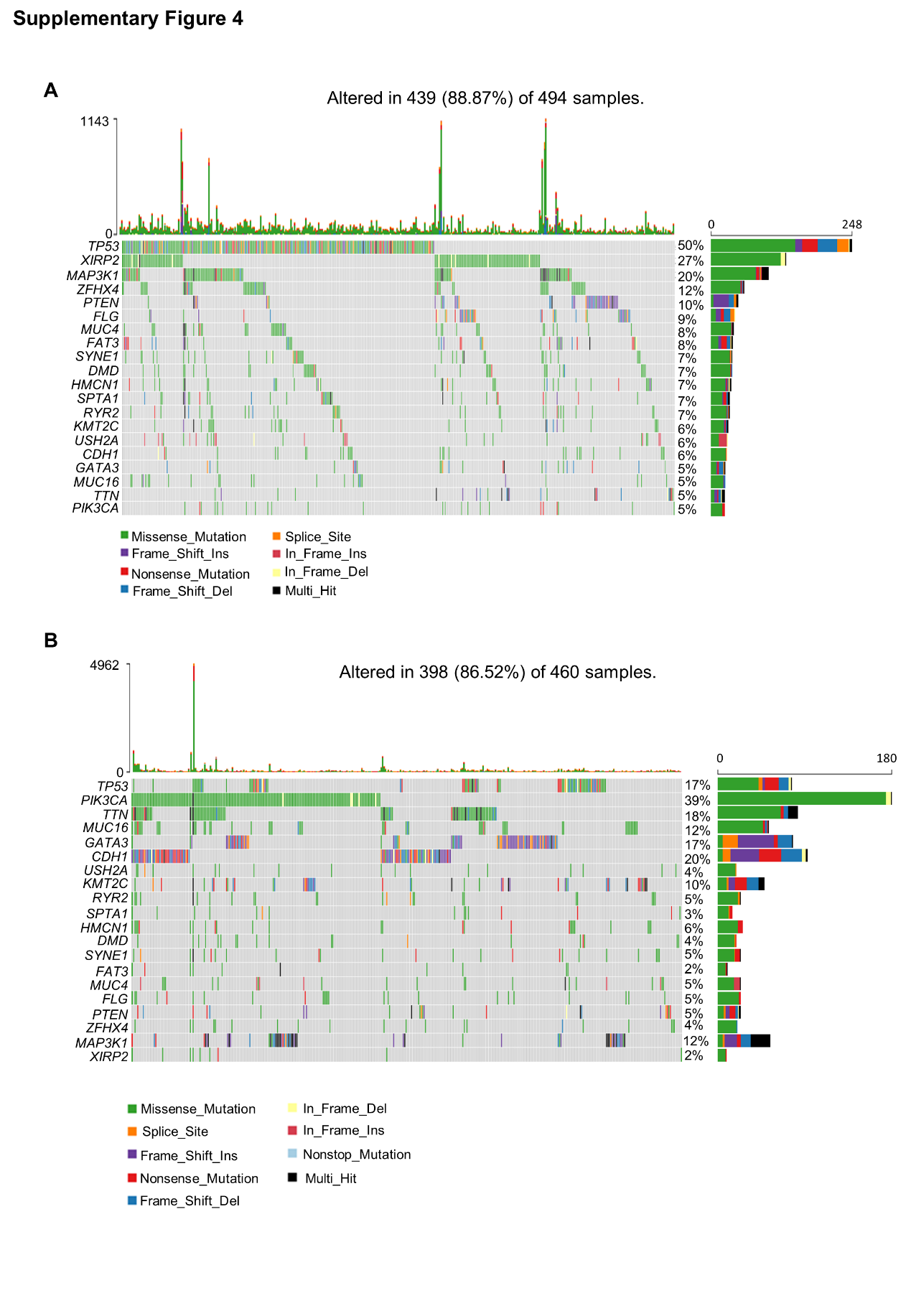


**Supplementary Figure 4.** Characteristics of lactate score and tumor somatic mutation. Waterfall plot of somatic mutation features of patients with high **(A)** and low **(B)** lactate scores. Each column represents an individual patient. The upper bar plot shows the TMB, and the number on the right indicates the mutation frequency of each gene. The right bar plot shows the proportion of each variant type.


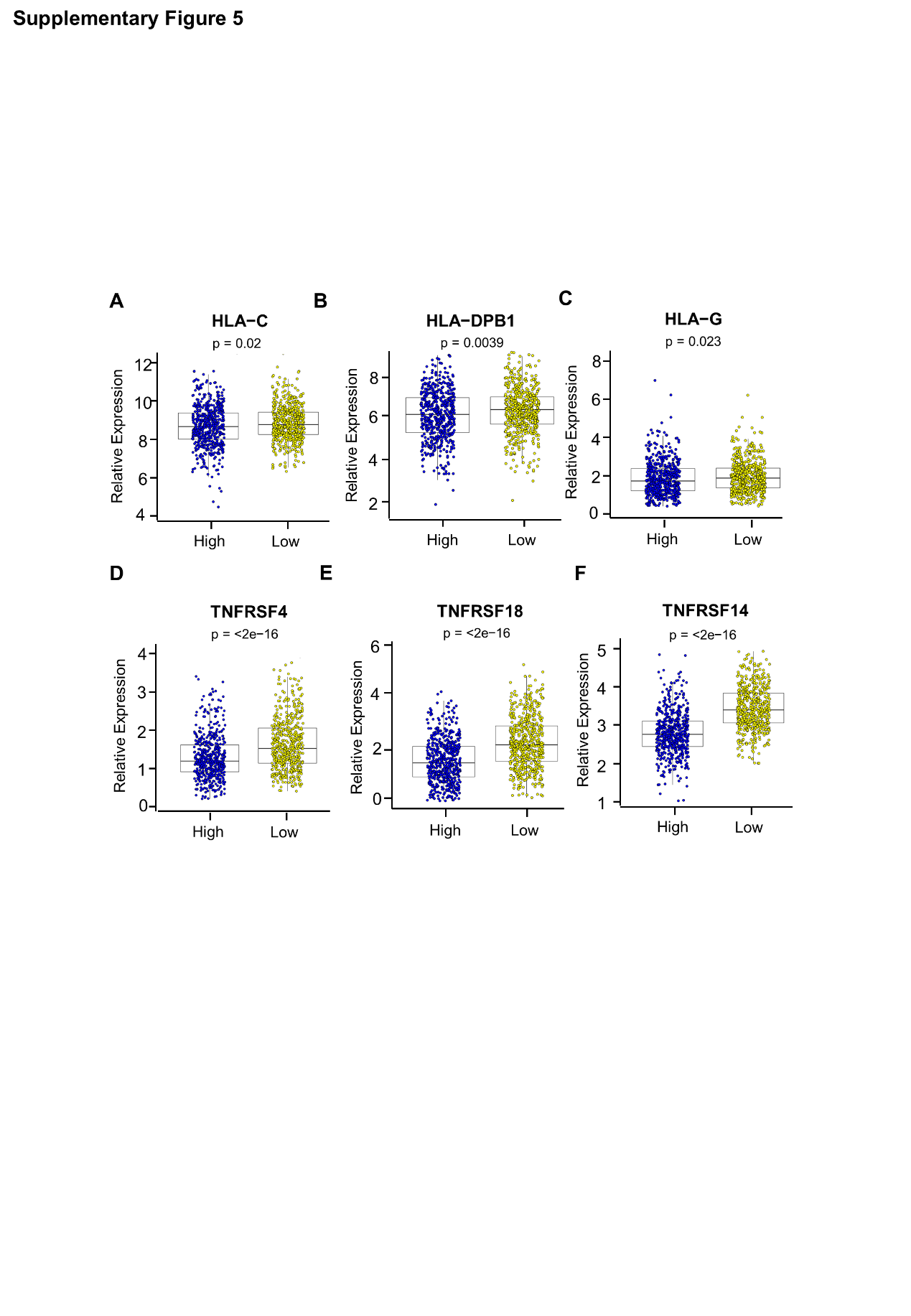


**Supplementary Figure 5.** Expression of immune molecules involved in T cell activation in the high and low lactate score groups.


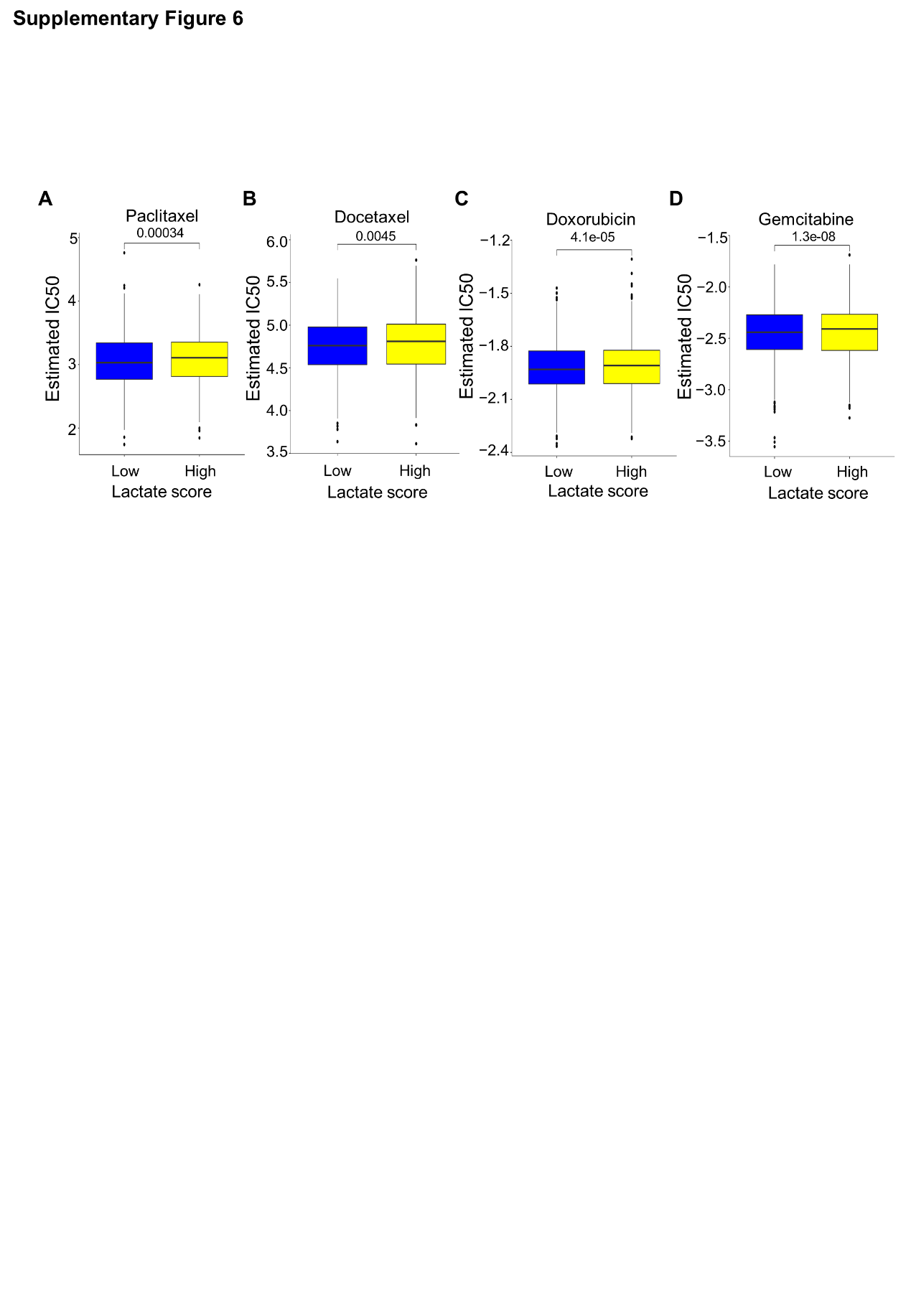


**Supplementary Figure 6**. Relationships between lactate score and chemotherapeutic sensitivity in BRCA.
